# Mapping the HLA-A24-restricted T-cell epitope peptide from a tumour-associated antigen HER2 / neu: possible immunotherapy for colorectal carcinomas

**DOI:** 10.1054/bjoc.2000.1547

**Published:** 2001-01-01

**Authors:** H Tanaka, T Tsunoda, I Nukaya, A Sette, K Matsuda, Y Umano, H Yamaue, K Takesako, H Tanimura

**Affiliations:** Department of Surgery II, Wakayama Medical School, Wakayama, 641-0012, Japan; Department of Surgery and Bioengineering, Advanced Clinical Research Center, Institute of Medical Science, The University of Tokyo, 4-6-1 Shirokane-dai, Minato-ku, Tokyo, 108-8639, Japan; Biotechnology Research Laboratories, Takara Shuzo Co. Ltd., Otsu, Shiga, 520-21, Japan

**Keywords:** CTL, epitope peptide, HER2 / neu, colorectal carcinoma

## Abstract

HER2 / neu is a potential antigen candidate for immunotherapy because of its correlation to a poor prognosis and high expressions in many kinds of epithelial tumours. Especially in the colorectal carcinomas, the higher expression of HER2 / neu is recognized in metastatic regions as well as in primary sites. Several CTL epitopes restricted by HLA-A2.1 and -A3 were identified so far, however epitopes restricted by HLA-A24, that is one of the most common allele in Japanese and Caucasians, have not been identified. In this paper, we showed identification of a CTL epitope peptide of HER2 / neu restricted by HLA-A24. HLA-A24 binding peptides selected by an analysis based on HLA-A24 binding motifs were determined for their binding affinities to HLA-A24 molecules. The peptide with a sequence of RWGLLLALL (position 8–16) named HE1 showed the highest affinity. We induced CTLs from CD8^+^cells of HLA-A24 healthy donors by stimulation with HE1-pulsed autologous dendritic cells. The CTLs showed cytotoxic activity against not only the peptide-pulsed target cells but also HLA-A24 colorectal tumour cell lines that endogenously overexpressed HER2 / neu. The antigen-specificity was confirmed by cold target inhibition assay using HE1-pulsed target cells. In summary, HER2 / neu peptide, RWGLLLALL, may contribute to the induction of antitumour immunity with the peptide-based immunotherapy for the colorectal carcinomas. © 2001 Cancer Research Campaign http://www.bjcancer.com


British Journal of Cancer (2001) 84(1), 94–99
© 2001 Cancer Research Campaign
doi: 10.1054/ bjoc.2000.1547, available online at http://www.idealibrary.com on IDEAL®

http://www.bjcancer.com

### Mapping the HLA-A24-restricted T-cell epitope peptide from a tumour-associated antigen HER2 / neu: possible immunotherapy for colorectal carcinomas

**H Tanaka[1], T Tsunoda[1,2], I Nukaya[3], A Sette[3], K Matsuda[1], Y Umano[1], H Yamaue[1], K Takesako[3] and H Tanimura[1]**

[1]Department of Surgery II, Wakayama Medical School, Wakayama, 641-0012, Japan; [2]Department of Surgery and Bioengineering, Advanced Clinical Research Center, Institute of Medical Science, The University of Tokyo, 4-6-1 Shirokane-dai, Minato-ku, Tokyo, 108-8639, Japan; [3]Biotechnology Research Laboratories, Takara Shuzo Co., Ltd. Otsu, Shiga 520-21, Japan

**Summary** HER2 / neu is a potential antigen candidate for immunotherapy because of its correlation to a poor prognosis and high expressions in many kinds of epithelial tumours. Especially in the colorectal carcinomas, the higher expression of HER2 / neu is recognized in metastatic regions as well as in primary sites. Several CTL epitopes restricted by HLA-A2.1 and -A3 were identified so far, however epitopes restricted by HLA-A24, that is one of the most common allele in Japanese and Caucasians, have not been identified. In this paper, we showed identification of a CTL epitope peptide of HER2 / neu restricted by HLA-A24. HLA-A24 binding peptides selected by an analysis based on HLA-A24 binding motifs were determined for their binding affinities to HLA-A24 molecules. The peptide with a sequence of RWGLLLALL (position 8–16) named HE1 showed the highest affinity. We induced CTLs from $CD8^+$ cells of HLA-A24 healthy donors by stimulation with HE1-pulsed autologous dendritic cells. The CTLs showed cytotoxic activity against not only the peptide-pulsed target cells but also HLA-A24 colorectal tumour cell lines that endogenously overexpressed HER2 / neu. The antigen-specificity was confirmed by cold target inhibition assay using HE1-pulsed target cells. In summary, HER2 / neu peptide, RWGLLLALL, may contribute to the induction of antitumour immunity with the peptide-based immunotherapy for the colorectal carcinomas. © 2001 Cancer Research Campaign http://www.bjcancer.com

**Keywords**: CTL; epitope peptide; HER2 / neu; colorectal carcinoma

Since the MAGE family's identification, many tumour-associated antigens (TAAs) have been discovered (van der Bruggen et al, 1991; Boon, 1993; Brichard et al, 1993; Kawakami et al, 1994; Boon and van der Bruggen 1996), and the approach to use them as targets of immunotherapy is now being rapidly developed. HER2 / neu is a 185-kD transmembrane glycoprotein with the tyrosine kinase activity, and its structure resembles that of the epidermal growth factor receptor (Coussens et al, 1985; Yamamoto et al, 1986). HER2 / neu is broadly amplified or overexpressed in many kinds of carcinomas, especially in adenocarcinomas that have not been confirmed as a target for immunotherapy (Slamon et al, 1989; Williams et al, 1991; Yoshino et al, 1994; Albino et al, 1995; Tsai et al, 1995; Lonn et al, 1996; Kapitanovic et al, 1997; Xia et al, 1997; Brossart et al, 1998). In addition, the degree of amplification is positively related to the poor prognoses of patients (Albino et al, 1995; Lonn et al, 1996; Kapitanovic et al, 1997) and their resistance to the anticancer chemotherapy (Tsai et al, 1995). In colorectal carcinomas, a higher expression of HER2 / neu is found in metastatic sites compared with primary regions (Kapitanovic et al, 1997). In other words, HER2 / neu is one of the attractive and expected antigen candidates for immunotherapy.

In order to use HER2 / neu as a target of CTLs, some epitopes restricted by HLA-A2 or -A3 were identified by some methods (Fisk et al, 1994, 1995; Peoples et al, 1995; Kono et al, 1998; Kawashima et al, 1999). However, to generalize the peptide-based immunotherapy, the identification of the epitopes restricted by other popular major histocompatibility (MHC) is required.

To establish the immunotherapy for the colorectal carcinoma that is still difficult to cure the patients in an advanced stage, the vaccine therapy with dendritic cells (DCs) and tumour antigen peptides might be an attractive and hopeful method (Romani et al, 1994; Morse et al, 1999). Herein, we have newly identified an epitope peptide of HER2 / neu restricted by HLA-A24, one of the most common alleles in Japanese and Caucasians (Kubo et al, 1994; Kondo et al, 1995; Date et al, 1996). And the CTLs induced by peptide-pulsed autologous DCs specifically killed HLA-A24-positive and HER2 / neu- overexpressing colorectal carcinoma cell lines.

### MATERIALS AND METHODS

### Cell lines

The TISI cells, human B-lymphoblastoid cell line expressing HLA-A24, were used for peptide-mediated cytotoxicity assays. The carcinoma cell line SKOV3 (HLA-A3 / A11, HER2 / $neu^+$) and its HLA-A24-transfected cell line SKOV3 / A24 were kindly provided by Epimmunce Inc (San Diego, CA). The colon carcinoma cell lines, HCT-15, HT29, and Colo320DM (all HLA-A24), were supplied by Shionogi Pharmaceuticals (Osaka, Japan). These cell lines were maintained in a tissue culture flask using RPMI 1640 containing HEPES and supplemented with antibiotics and 10% heat-inactivated fetal calf serum (Gibco BRL). The expression of HER2 / neu proteins was determined by flowcytometric

*Received 6 March 2000*
*Revised 13 September 2000*
*Accepted 20 September 2000*

*Correspondence to:* T Tsunoda analysis using the anti-HER2 / neu monoclonal antibody (Bender MedSystems, Austria).

### Synthetic peptides

Peptides were synthesized according to the standard solid phase synthesis method and purified by reversed phase HPLC. The purity ( > 90%) and the identity of the peptides were determined by analytical HPLC and mass spectrometry analysis, respectively. Peptides were dissolved in dimethylsulfoxide at 20 mg $ml^{-1}$ and stored at –20˚C.

### MHC binding assays

The binding capacity of peptides to HLA-A24 molecules was measured based on the inhibition of binding of a radiolabelled standard peptide to purified MHC molecules as described by Kondo et al (1995). Briefly, various concentrations of the test peptides were incubated with $^{125}$I-labelled standard peptide (sequence AYIDNYNKF) and purified and detergent-solubilized HLA-A24 molecules in the presence of a mixture of protease inhibitors and β-2-microglobulin (Scripps Laboratories, San Diego, CA). The percentage of HLA-A24-bound radioactivity was determined by gel filtration and the concentration of the test peptides that inhibited 50% of the binding of the labelled peptide (IC50) were calculated respectively.

### Primary CTL induction cultures

Tissue culture-derived DCs were used as APC to trigger CTL responses using MHC-binding peptides. DCs were generated in vitro as described (Tsai et al, 1997; Nukaya et al, 1999). Briefly, PBMC isolated from a normal volunteer (HLA-A24 / A2) by Ficoll-Paque (Pharmacia, Piscataway, NJ) were separated by adherence to a plastic tissue culture flask so as to enrich them for the monocyte fraction. The monocyte-enriched population was cultured in the presence of 1000 U $ml^{-1}$ of GM-CSF (Genzyme) and 2000 U $ml^{-1}$ of IL-4 (Genzyme) in RPMI 1640 containing HEPES and supplemented with 2 mM of L-glutamine, 1 mM of sodium pyruvate and 0.1 mM of non-essential amino acids solution, 5% heat-inactivated AB human serum (all reagents from BioWhittaker, Walkersville, MD), and antibiotics (RPMI / 5% HS). After 7 days in the culture, the cytokine-generated DCs were pulsed with 40 μg $ml^{-1}$ of MHC-binding peptides in the presence of 3 μg $ml^{-1}$ of β2-microglobulin for 4 h at 20˚C in PBS with 1% bovine serum albumin. These peptide-pulsed DCs were then irradiated (5500 rads) and mixed at a 1:20 ratio with autologous $CD8^+$ T cells, obtained by positive selection with Dynabeads M-450 CD8 (Dynal, Lake Success, NY) and Detachabead (Dynal). These cultures were set up in 48-well plates; each well contained $0.25 \times 10^5$ peptide-pulsed DC, $5 \times 10^5$ $CD8^+$ cells and 10 ng $ml^{-1}$ IL-7 (Genzyme) in 0.5 ml of RPMI / 5% HS. One day later, these cultures were supplemented with IL-10 (R&D Systems, Minneapolis, MN) to a final concentration of 10 ng $ml^{-1}$. On days 7 and 14, the T cell cultures were further restimulated with the autologous peptide-pulsed adherent APC. The adherent cells were prepared each times. To prepare the adherent APC, $2 \times 10^6$ irradiated autologous PBMC in 0.5 ml of RPMI / 5% HS were added to each well of 48-well plates. After incubation at 37˚C for 1.5 h, the non-adherent cells were washed off, and the adherent cells were incubated for 2 h with 0.2 μg $ml^{-1}$ of peptide and 3 μg $ml^{-1}$ of β2-microglobulin in a final volume of 0.25 ml of RPMI / 5% HS per well. Supernatants of the responder cultures were aspirated and RPMI / 5% HS was added to the total volume of 0.5 ml per well. After removal of the excess peptide from the wells of the adherent APC, the responder cultures were transferred to the corresponding wells containing peptide-pulsed APC. Each individual well was restimulated separately and on the day following each restimulation, a final concentration of 10 ng $ml^{-1}$ of IL-10 was added to each culture. The cultures were fed every 2 to 3 days with RPMI / 5% HS containing 10 U $ml^{-1}$ of IL-2 (Shionogi, Osaka, Japan). Cytotoxicity was tested after 2 and 4 rounds of peptide stimulation on day 21 and day 36 using peptide-pulsed TISI cells. Responder cells in the positive wells for peptide eradication were expanded as described below and tested for their cytotoxicity to SKOV3 / A24 tumour cells, which naturally produce and process the HER2 / neu molecule.

### CTL cytotoxicity assays

Adherent target cells were detached from tissue culture flasks with trypsin-EDTA. All cells were labelled with 100 μCi of $^{51}$Cr (Dupont) per $3 \times 10^6$ cells for 1 h at 37˚C. Peptide-pulsed targets were prepared by incubating the cells at $5 \times 10^5$ cells $ml^{-1}$ with 10 μg $ml^{-1}$ of the peptide for 16 h at 37˚C. Target cells were washed by centrifugation and mixed with effectors in a final volume of 0.2 ml in round-bottom microtitre plates. The plates were centrifuged (2 min at 400 ***g***) to increase cell-to-cell contact and placed in a $CO_2$ incubator at 37˚C. After 4 h of incubation, 0.1 ml of the supernatant was removed from each well and the radioactivity was determined in a gamma counter. In the initial screening for the presence of cytolytic activity in each well of the 48-well minicultures, the effector cells were routinely not counted. However, sample counting of these cells indicated that effector to target (E:T) ratios in these assays were in the range of 10:1 to 1:1. To eliminate any non-specific lysis due to NK-like effectors, the cytolytic activity was tested in the presence of a 30-fold excess of unlabelled K562 cells. Antigen specificity was confirmed by cold target inhibition experiments, which utilized unlabelled TISI cells that were pulsed with peptide (10 μg $ml^{-1}$ for 16 h at 37˚C) to compete for the recognition of $^{51}$Cr-labelled HCT-15 tumour cells.

The percentage of specific cytotoxicity was determined by calculating the percentage of specific $^{51}$Cr release according to the following formula: (cpm of the test sample release – cpm of the spontaneous release) / ((cpm of the maximum release – cpm spontaneous release)) × 100. The spontaneous release was determined by incubating the target cells alone, in the absence of effectors, and the maximum release was obtained by incubating the targets with 1N-HCl. All determinants were done in duplicates, and the SEMs were consistently below 10% of the value of the mean.

### CTL expansion procedure

Responder cells or CTL lines were expanded in tissue culture following a method similar to the one described by Riddell et al (Walter et al, 1995; Riddel et al, 1996). A total 5 × 104 CTL were resuspended in 25 ml of RPMI / 5% HS with 25 × 106 irradiated (3300 rads) PBMC and 5 × 106 irradiated (8000 rads) Epstein-Barr virus-transformed B-lymphoblastoid cell line EHM cells (HLA-A3 / 3) in the presence of 30 ng $ml^{-1}$ of anti-CD3 monoclonal antibodies. One day after initiating the cultures, 120 lU $ml^{-1}$ of IL-2 were added to the cultures. The cultures were fed with fresh RPMI / 5% HS containing 30 IU $ml^{-1}$ of IL-2 on days 5, 8

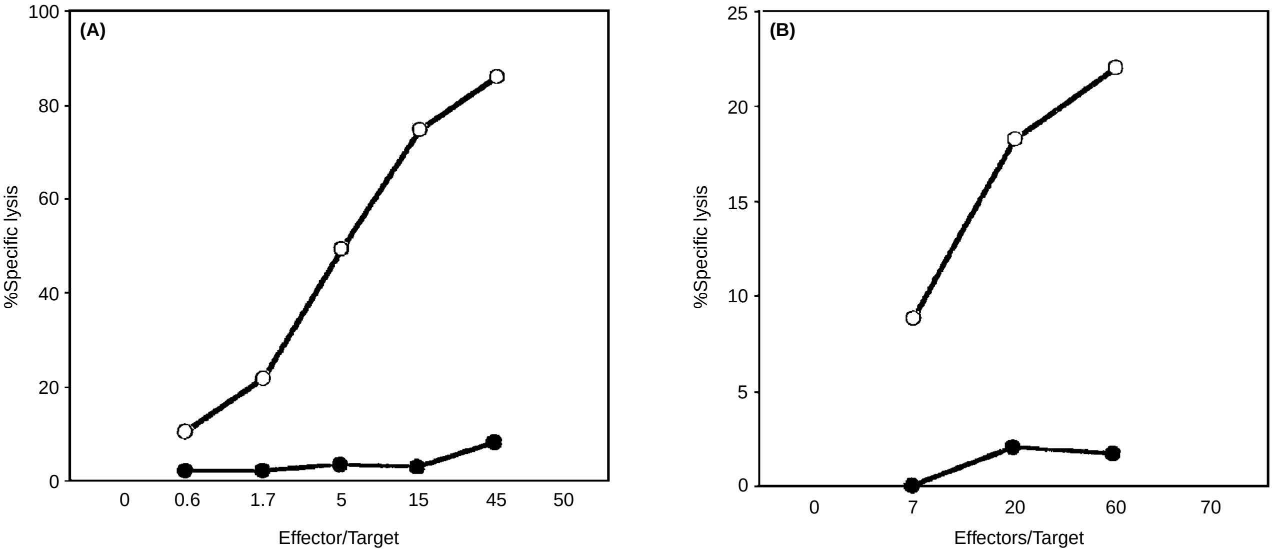

**Figure 1** Cytotoxic activity against peptide-pulsed target cells and ovarian carcinoma cell lines expressing HER2 / neu. The CTLs expanded after 4 cycles of restimulations with peptides were tested for their cytotoxic activity. (**A**) The recognition of peptide (HE1)-pulsed TISI by the CTSs.: ○, TISI pulsed with peptide (HE1); ●, TISI without peptide. (**B**) Recognition of HLA-A24 transfected SKOV3, ovarian cancer cell line that overexpresses HER-2/neu: , ●, SKOV3 (ovarian cancer, A3/A11, HER2/neu$^+$); ○, SKOV3/A24 (ovarian cancer, A24$^+$, HER2/neu$^+$)

and 11 and were split if the T-cell concentration reached numbers > 1.5 × 106 ml-1· On average, approximately 1 to 2 × 107 CTL were obtained by days 12–14.

### RESULTS

### Determination of the HLA-A24 binding peptides from HER-2 / neu

The amino acid sequence of HER2 / neu (Yamamoto et al, 1986) was scanned for peptides having a length of 9 or 10 amino acids, containing the HLA-A24 binding motif (Y, F or W at position 2 and F, L, I, W or M at the C-terminus) (Kubo et al, 1994). Of a total of 15 motif-including peptides tested for HLA-A24 binding, 5 peptides were found to bind to the purified HLA-A24 molecules with a high affinity ($IC_{50}$ < 50 nM, Table 1) and 10 bound to the HLA-A24 molecules with an intermediate to low range of affinities ($IC_{50}$ of 50–500 nM, data not shown).

### CTLs induced with HE1 peptide-pulsed DC indicated peptide-specific and HLA-A24 restricted cytotoxicity

We selected the peptide RWGLLLALL called HE1, (located at position 8 to 16 from the N-terminus of HER2 / neu protein), because it showed the distinctive binding to the purified HLA-A24 molecules. This peptide was studied for its capacity to elicit the CTL response by primary in vitro immunization of CD8$^+$ T cells stimulated with the peptide-pulsed autologous DCs using PBMCs from two donors. After 4 rounds of the restimulation with antigen, several wells showed the positive cytotoxic activity. We chose the CTLs in one well which showed the strongest peptide-specific cytotoxic activity in each donor. After in vitro expansion, the CTLs from one donor lysed HE1-pulsed TISI cells but not unpulsed TISI cells which HE1 was not pulsed (% specific lysis at E / T = 5, 50% for TISI pulsed with peptide and 4% for TISI without peptide, Figure 1A). The cytotoxic activity of the CTL against HE1-pulsed TISI could be observed even at low E / T ratio. The CTLs lysed HER2 / neu-overexpressing ovarian carcinoma cell line SKOV3 / A24 but not its parent cell line SKOV3 (% specific

**Table 1** Binding affinity of HER-2/neu peptides to HLA-A24 molecules

| Code | Position | Peptide sequence | $IC_{50}$ (nM) |
|---|---|---|---|
| HE1 | 8–16 | RWGLLLALL | 4.5 |
| HE2 | 780–788 | PYVSRLLGI | 34 |
| HE3 | 952–960 | VYMIMVKCW | 36 |
| HE4 | 440–448 | AYSLTLQGL | 45 |
| HE5 | 907–915 | SYGVTVWEL | 48 |

The HER[8–16]: RWGLLLALL (abbreviated HE1) showed the strongest binding to the purified HLA-A24 molecules.

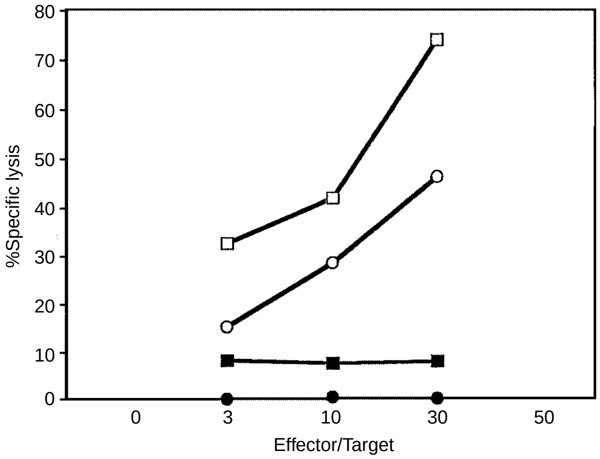

**Figure 2** Expression of HER2 / neu for colorectal carcinoma cell lines by FACS. The dotted line indicates the secondary antibody alone (FITC conjugated controlled antibody). The black line indicates that anti-HER2 / neu monoclonal antibody plus the secondary antibody

© 2001 Cancer Research Campaign

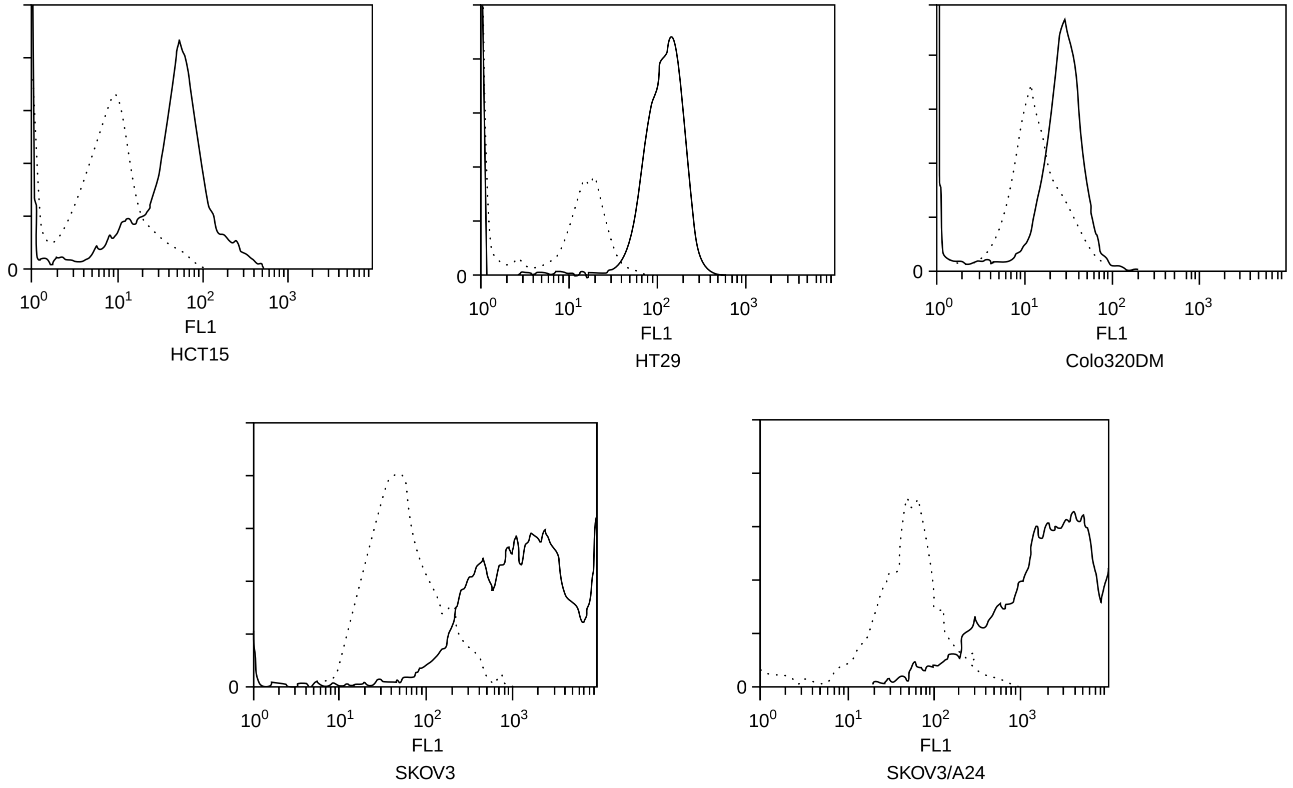

**Figure 3** Tumour reactivity and antigen specificity of HLA-A24-restricted HE1-specific CTL. The HE1-specific CTL expanded after 4 cycles of restimulations with peptide was used as effector cells to test the cytotoxic activity of the following target cell lines: □, HCT-15 (colon carcinoma, A24⁺, HER2/neu⁺); ○, HT29 (colon carcinoma, A24⁺, HER2 / neu⁺); ■, SKOV3 (ovarian carcinoma, A24-, HER2/neu⁺); ●, Colo320DM (colon carcinoma, A24⁺, HER2 / neu-)

lysis at E / T = 20, 19% and 4%, respectively) (Figure 1B). These results indicate that the CTL induced by HE-1 peptide showed cytotoxicity with peptide-specificity and HLA-A24-restricticity.

### Cytotoxic activity of the CTLs against colorectal carcinoma cell lines in HER2 / neu specific and HLA-A24-restricted manner

To clarify the cytotoxic activity of the CTLs against colorectal carcinoma cell lines that endogenously express HER2 / neu, we used 3 colorectal carcinoma cell lines. We tested for expression of HER2 / neu by way of flow cytometry. Expression of HER2 / neu was shown to be stronger at metastatic sites of colorectal tumours than at primary lesions (Kapitanovic et al, 1997). Colorectal carcinoma cell lines, HCT 15 and HT29, expressed HER2 / neu highly, whereas Colo320DM did very weakly (Figure 2). Then, cytotoxic activity of the CTLs induced by HE1 against these cell lines was investigated. The CTLs showed the distinct cytotoxic activity against HLA-A24-positive and HER2 / neu-overexpressing cell lines, HT29 and HCT-15 (% specific lysis at E / T = 30, 74% and 46%, respectively, Figure 3). On the other hand, the cytotoxic activity of the CTLs against Colo320DM and SKOV3 that lacked either HLA-A24 or strong expression of HER2 / neu, was very weak or absent, 8% and 0%, respectively (Figure 3). Furthermore, the cytotoxic activity against HCT-15 was inhibited by cold targets (peptide-pulsed TISI), but not by TISI without peptide, showing that the cytotoxicity was specific to HER2 / neu (Table 2).

**Table 2** Antigen specificity of the CTLs by cold target inhibition assay

| Exp. | Inhibitor/Target | Inhibitor (%) | | % Inh. |
|---|---|---|---|---|
| | | TISI alone | TISI + HE1 | |
| 1 | 3 | 18.3 | 17.9 | 2.2 |
| | 10 | 18.4 | 12.8 | 30.4 |
| 2 | 30 | 17.1 | 7.4 | 56.7 |

As a hot target, HCT15 (HER2 / neu and HLA-A24 positive) cells were labelled with $^{51}Cr$ and mixed with TISI (HLA-A24 positive LCL) pulsed with HE1 and TISI without peptide as cold targets. The results were indicated as a percentage of specific lysis at the E / T ratio of 10.

### DISCUSSION

Adenocarcinomas including colorectal carcinomas have not been considered as the suitable candidates for antitumour immunotherapy because of their low immunogenicity, compared with malignant melanoma (Mukherji et al, 1995; Hu et al, 1996; Rosenberg et al, 1998). On the other hand, a large number of patients die of various kinds of adenocarcinomas, therefore, the antigens expressed in adenocarcinomas are eagerly expected to be the targets for immunotherapy.

Especially HER2 / neu is a TAA that is expressed in various kinds of carcinomas including breast, ovary, and colorectal carcinoma (Slamon et al, 1989; Williams et al, 1991; Yoshino et al, 1994; Albino et al, 1995; Tsai et al, 1995; Lonn et al, 1996; Kapitanovic et al, 1997; Xia et al, 1997; Brossart et al, 1998). In addition, this antigen has some characteristics that its strong

*© 2001 Cancer Research Campaign* 

expression at the primary site of a patient has related to the poor prognosis (Lonn et al, 1996; Xia et al, 1997), and that the expression of metastatic sites is stronger than the primary sites (Kapitanovic et al, 1997). From these facts, HER2 / neu has been considered to be one of the potential tumour antigens not only for CTL therapy but also for antibody therapy (Bacus et al, 1992; Baselga et al, 1998; Ward et al, 1999).

Some HLA-A2 or -A3-restricted peptides of HER2 / neu that elicit the CTL activity have identified so far (Fisk et al, 1994, 1995; Peoples et al, 1995; Kono et al, 1998; Kawashima et al, 1999), however the epitope peptide that is restricted by HLA-A24 has not been identified yet. HLA-A24 is one of the most popular HLA allele in Japanese ( > 60%) and also in Caucasian (Kubo et al, 1994; Kondo et al, 1995; Date et al, 1996). Moreover, most of their microallele consist of HLA-A*2402. Thus we have little necessity to consider the variations of the motifs between microalleles and this is a distinctive advantage compared with other popular HLA allele like HLA-A2.

In this study, we identified the HLA-A24-restricted epitope peptide of HER2 / neu (Kapitanovic et al, 1997) (HE1: RWGLLLALL) that elicited the antigen-specific cytotoxicity in HLA-A24-restricted manner. At first, we investigated the affinity to solubulized HLA-A24 molecules of over 20 peptides having binding-motifs to HLA-A24, considering that the peptides having high affinity to HLA molecules could be presented by APCs more frequently. Then we tested 5 high-binding peptides shown in Table 1 for the CTL induction activity, with the result that only the CTLs stimulated with HE1 showed the cytotoxicity against the peptide-pulsed TISI (data not shown). Thus we chose the HE1 to the further analyses. In the in vitro primary induction with DCs, CTL lines induced by HE1 showed the considerable cytotoxic activity. The generated CTL lines showed specific cytotoxicity not only against the peptide-pulsed target cells but also against HLA-A24 colorectal carcinoma cell lines overexpressing HER2 / neu. Furthermore, they did not show the cytotoxic activity against the cell lines that lacked either of them. In the cold target inhibition assay, the cytotoxicity against a colon carcinoma cell line HCT-15 was inhibited by the HE1-pulsed TISI and was not by TISI without peptide. Based on these results, we finally determined the HE1 peptide as one of an epitope peptide restricted by HLA-A24.

In this study, it is clear that DCs had a very important role as APCs for inducing peptide-specific CTLs. DCs have been considered as efficient tools to induce the immune responses against cancers even in vivo (Reeves et al, 1996; Hart, 1997; Nestle et al, 1998). We showed HER2 / neu-specific CTLs could be induced by autologous DCs and HE1. This result will be helpful for clinical applications of colorectal carcinoma vaccines in the near future. In order to promote the clinical response for cancer vaccine, it is important to map as many epitope peptides as possible. Clinical studies will be necessary to define the optimal setting, maybe the situation of minimal residual disease or adjuvant therapy, as well as adequate vaccination procedures and antigen concentrations.

### REFERENCES

Albino AP, Jaehne J, Altorki N, Blundell M, Urmacher C, Lauwers G, Niedzwiecki D and Kelsen DP (1995) Amplification of HER-2 / neu gene in human gastric adenocarcinomas: colleration with primary site. *Eur J Surg Oncol* **21**: 56–60

Bacus SS, Stancovski I, Huberman E, Chin D, Hurwitz E, Mills GB, Ullrich A, Sela M and Yarden Y (1992) Tumor-inhibitory monoclonal antibodies to the HER-2 / neu receptor induce differentiation of human breast cancer cells. *Cancer Res* **52**: 2580–2589

Baselga J, Norton L, Albanell J, Kim YM and Mendelsohn J (1998) Recombinant humanized anti-HER-2 / neu antibody (Herceptin) enhances the antitumor activity of paclitaxel and doxolubicin against HER-2 / neu overexpressing human breast cancer xenographts. *Cancer Res* **58**: 2825–2831

Boon T (1993) Tumor antigens recognized by cytotoxic T lymphocytes: Present perspectives for specific immunotherapy. *Int J Cancer* **54**: 177–180

Boon T and van der Bruggen P (1996) Human tumor antigen recognized by T lymphocytes. *J Exp Med* **183**: 725–729

Brichard V, Van Pel A, Wolfel T, Wolfel C, De-Plaen E, Lethe B, Coulier P and Boon T (1993) The tyrosinase gene codes for an antigen recognized by autologous cytolytic T lymphocytes on HLA-A2 melanomas. *J Exp Med* **178**: 489–495

Brossart P, Stuhler G, Flad T, Stevanovic S, Rammensee HG, Kanz L and Brugger W (1998) Her-2 / neu-derived peptides are tumor-associated antigens expressed by human renal cell and colon carcinoma lines and are recognized by in vitro induced specific T lymphocytes. *Cancer Res* **58**: 732–736

Celis E, Tsai V, Crimi C, DeMars R, Wentworth PA, Chesnut RW, Grey HM, Sette A and Serra HM (1994) Induction of anti-tumor cytotoxic T lymphocytes in normal humans using primary cultures and synthetic peptide epitopes. *Proc Natl Acad Sci USA* **91**: 2105–2109

Coussens L, Yang-Feng TL, Liao YC, Chen E, Gray A, McGrath J, Seeburg PH, Libermann TA, Schlessinger J, Francke U, Levinson A and Ullrich A (1985) Tyrosine kinase receptor with extensive homology to EGF receptor shares chromosomal location with neu oncogene. *Science*, **230**: 1132–1139

Date Y, Kimura A, Kato H and Sasazuki T (1996) DNA typing of the HLA-A gene: population study and identification of four new alleles in Japanese. *Tissue Antigens* **47**: 93–101

Fisk B, Chesak B, Ioannides MG, Wherton JT and loannides CG (1994) Sequence motifs of human HER-2 proto-oncogene important for peptide binding to HLA-A2. *Int J Oncol* **5**: 51–62

Fisk B, Blevins TL, Wherton JT and Ioannides CG (1995) Identification of an immunodominant peptide of HER-2 / neu protoncogene recognized by ovarian tumor-specific cytotoxic T lymphocyte lines. *J Exp Med* **181**: 2109–2117

Hart DNJ (1997) Dendritic cells: unique leucocyte populations which control the primary immune response. *Blood*, **90**: 3245–3287

Hu X, Chakraborty NG, Sporn JR, Kurtzman SH, Ergin MT, Mukherji BH (1996) Enhancement of cytolytic T lymphocyte precursor frequency in melanoma patients following immunization with the MAGE-1 peptide loaded antigen presenting cell-based vaccine. *Cancer Res* **56**: 2479–2483

Kapitanovic S, Radšoevic S, Kapitanovic M, Andelinovic Š, Ferencic Z, Tavassoli M, Rimorac D, Sonicki Z, Spaventi Š, Pavelic K and Spaventi R (1997) The expression of p185HER-2 / neu correlates with the stage of diseases and survival in colorectal cancer. *Gastroenterology* **112**: 1103–1113

Kawakami Y, Eliyahu S, Sakaguchi K, Robbins PF, Rivoltini L, Yannelli JR, Appella E and Rosenberg SA (1994) Identification of the immunodominant peptides of the MART-1 human melanoma antigen recognized by the majority of HLA-A2-restricted tumor infiltrating lymphocytes. *J Exp Med* **180**: 347–352

Kawashima I, Tsai V, Southwood S, Takesako K, Sette A and Celis E (1999) Identification of HLA-A3-restricted cytotoxic T lymphocyte epitopes from carcinoembryonic antigen and HER-2 / neu by primary in vitro immunization with peptide pulsed dendritic cells. *Cancer Res* **59**: 431–435

Kondo A, Sidney J, Southwood S, Del Guercio MF, Appella E, Sakamoto H, Celis E, Grey HM, Chesnut RW, Kubo RT and Sette A (1995) Prominent roles of secondary anchor residues in peptide binding to HLA-A24 human class I molecules. *J Immunol* **155**: 4307–4312

Kono K, Rongcun Y, Charo J, Ichihara F, Celis E, Sette A, Appella E, Sekikawa T, Matsumoto Y and Kiessling R (1998) Identification of HER2 / neu-derived peptide epitopes recognized by gastric cancer-specific cytotoxic lymphocytes. *Int J Cancer* **78**: 202–208

Kubo RT, Sette A, Grey HM, Appella E, Sakaguchi K, Zhu NZ, Arnott D, Sherman N, Shabanowitz J, Michel H, Bodnar WM, Davis TA and Hunt DF (1994) Definition of specific peptide motifs for four major HLA-A alleles. *J Immunol* **152**: 3913–3924

Lönn U, Lönn S, Nilsson B and Stenkvist B (1996) Prognostic significance of c-erb-B2 amplification in fine needle biopsies of breast cancer patients not operated at diagnosis. *Breast Cancer Res and Treat* **39**: 213–220

Morse MA, Coleman RE, Akabani G, Niehaus N, Coleman D and Lyerly HK (1999) Migration of human dendritic cells after injection in patients with metastatic malignancies. *Cancer Res* **59**: 56–58

 © 2001 Cancer Research Campaign

Mukherji B, Chakraborty NG, Yamasaki S, Okino T, Yamase H, Sporn JR, Kurtzman SK, Ergin MT, Ozols J, Meehan J and Mauri F (1995) Induction of antigen-specific cytolytic T cells in situ in human melanoma by immunization with synthetic peptide-pulsed antologous antigen presenting cells. *Proc Natl Acad Sci USA* **92**: 8078–8082

Nestle FO, Alijagic S, Gilliet M, Sun Y, Grabbe S, Dummer R, Burg G and Schadendorf D (1998) Vaccination of melanoma patients with peptideor tumor lysate-pulsed dendritic cells. *Nature Med* **3**: 328–332

Nukaya I, Yasumoto M, Iwasaki T, Ideno M, Sette A, Celis E, Takesako K and Kato I (1999) Identification of HLA-A24 epitope peptide of carcinoembryonic antigen that induce tumor-reactive cytotoxic T lymphocyte. *Int J Cancer* **80**: 92–97

Peoples GE, Goedegebuure PS, Smith R, Linehan DC, Yoshino I and Eberlein TJ (1995) Breast and ovarian cancer specific cytotoxic T lymphocytes recognize the same HER2 / neu-derived peptide. *Proc Natl Acad Sci USA* **92**: 432–436

Reeves ME, Royal RE, Lam JS, Rosenberg SA and Hwu P (1996) Retroviral transduction of human dendritic cells with a tumor-associated antigen gene. *Cancer Res* **56**: 5672–5677

Riddel SR, Elliott M, Lewinsohn DA, Gilbert MJ, Wilson I, Manley SA, Lupton SD, Overell RW, Retnolds TC, Corey L and Greenberg PD (1996) T-cell mediated rejection of gene-modified HIV-specific cytotoxic T lymphocytes in HIV-infected patients. *Nature Med* **2**: 216–223

Romani N, Gruner S, Brang D, Kämpgen E, Lenz A, Trockenbacher B, Konwalinka G, Fritsch PO, Steinman RM and Schuler G (1994) Proliferating dendritic cell progenitors in human blood. *J Exp Med* **180**: 83–93

Rosenbeg SA, Yang JC, Schwartzentruber DJ, Hwu P, Marincola FM, Topalian SL, Restifo NP, Dudey ME, Schwarz SL, Spiess PJ, Wunderlich JR, Parkhurst MR, Kawakami Y, Seipp CA, Einhorn JH and White DE (1998) Immunologic and therapeutic evaluation of a synthetic peptide vaccine for the treatment of patients with metastatic melanoma. *Nature Med* **4**: 321–327

Slamon DJ, Godolphin W, Jones LA, Holt JA, Wong SG, Keith DE, Levin WJ, Stuart SG, Udove J, Ullrich A and Press MF (1989) Studies of the HER-2 / neu proto-oncogene in human breast and ovarian cancer. *Science* (Washington DC) **244**: 707–712

Tsai CM, Yu D, Chang KT, Wu LH, Peng RP, Ibrahim NK and Hung MC (1995) Enhanced chemoresistance by elevation of $p185^{neu}$ levels in HER-2 / neu-transfected human lung cancer cells. *J Natl Cancer Inst* **87**: 682–684

Tsai V, Southwood S, Sidney J, Sakaguchi K, Kawakami Y, Appella E, Sette A and Celis E (1997) Identification of subdominant CTL epitopes of the GP100 melanoma-associated tumor antigen by primary in vitro immunization with peptide-pulsed dendritic cells. *J Immunol* **158**: 1796–1802

Tsang KY, Zaremba S, Nieroda CA, Zhu MZ, Hamilton JM and Schlom J (1995) Generation of human cytotoxic T cell specific for human carcinoembryonic antigen epitopes from patients immunized with recombinant vaccinia-CEA vaccine. *J Natl Cancer Inst* **87**: 982–990

van der Bruggen P, Traversari C, Chomez P, Lurquin C, De-Plaen E and Boon T (1991) A gene encoding an antigen recognized by cytolytic T lymphocytes on a human melanoma. *Science* **254**: 1643–1647

Walter EA, Greenberg PD, Gilbert MJ, Finch RJ, Watanabe KS, Thomas ED and Riddel SR (1995) Reconstitution of cellular immunity against cytomegalovirus in recipients of allogeneic bone marrow transfer of T-cell clones from the donor. *N Engl J Med* **333**: 1038–1044

Ward RL, Hawkins NJ, Coomber D and Disis ML (1999) Antibody immunity to the HER-2 / neu oncogene protein in patients with colorectal cancer. *Hum Immun* **60**: 510–515

Williams TM, Weiner DB, Greene MI and Maguire HC Jr (1991) Expression of c-erb-B2 in human pancreatic adenocarcinomas. *Pathobiology* **59**: 46–52

Xia W, Lau YK, Zhang HZ, Liu AR, Li L, Kiyokawa N, Clayman GL, Katz RL and Hung MC (1997) Strong correlation between c-erbB-2 overexpression and overall survival of patients with oral squamous cell carcinoma. *Clin Can Res* **3**: 3–9

Yamamoto T, Ikawa S, Akiyama T, Semba K, Nomura N, Miyajima N, Saito T and Toyoshima K (1986) Similarity of protein encoded by the human c-erb-B-2 gene to epidermal growth factor receptor. *Nature* (Lond) **319**: 230–234

Yoshino I, Peoples GE, Goedegebuure PS, Maziarz R and Eberlein TJ (1994) Association of HER2 / neu expression with sensitivity to tumor-specific CTL in human ovarian cancer. *J Immunol* **152**: 2393–2400

*© 2001 Cancer Research Campaign* 
